# Synonymous alterations of cancer-associated *Trp53* CpG mutational hotspots cause fatal developmental jaw malocclusions but no tumors in knock-in mice

**DOI:** 10.1371/journal.pone.0284327

**Published:** 2023-04-13

**Authors:** Richard J. Epstein, Frank P. Y. Lin, Robert A. Brink, James Blackburn

**Affiliations:** 1 University of New South Wales, St Vincent’s Hospital Campus, Sydney, Australia; 2 Garvan Institute of Medical Research, Sydney, Australia; 3 Centre for Clinical Genomics, The Kinghorn Cancer Centre, Sydney, Australia; Virginia Commonwealth University, UNITED STATES

## Abstract

Intragenic CpG dinucleotides are tightly conserved in evolution yet are also vulnerable to methylation-dependent mutation, raising the question as to why these functionally critical sites have not been deselected by more stable coding sequences. We previously showed in cell lines that altered exonic CpG methylation can modify promoter start sites, and hence protein isoform expression, for the human *TP53* tumor suppressor gene. Here we extend this work to the in vivo setting by testing whether synonymous germline modifications of exonic CpG sites affect murine development, fertility, longevity, or cancer incidence. We substituted the DNA-binding exons 5–8 of *Trp53*, the mouse ortholog of human *TP53*, with variant-CpG (either CpG-depleted or -enriched) sequences predicted to encode the normal p53 amino acid sequence; a control construct was also created in which all non-CpG sites were synonymously substituted. Homozygous *Trp53*-null mice were the only genotype to develop tumors. Mice with variant-CpG *Trp53* sequences remained tumor-free, but were uniquely prone to dental anomalies causing jaw malocclusion (p < .0001). Since the latter phenotype also characterises murine Rett syndrome due to dysfunction of the trans-repressive MeCP2 methyl-CpG-binding protein, we hypothesise that CpG sites may exert non-coding phenotypic effects via pre-translational cis-interactions of 5-methylcytosine with methyl-binding proteins which regulate mRNA transcript initiation, expression or splicing, although direct effects on mRNA structure or translation are also possible.

## Introduction

The CpG dinucleotide represents a masterstroke of adaptive evolution [1–3], combining as it does the stabilization of Watson-Crick base pairs via its triple hydrogen-bonded structure [4–6] with destabilization of sequence fidelity via a susceptibility to cytosine methylation that in turn permits mutagenic deamination to thymine [7–9]. Even without this adaptive role, the informatic content of CpG sites exceeds that of amino acid coding alone, given that methylation of CpG-cytosines in canonical B-DNA regulates conformational changes in nucleosomes which affect gene transcription, duplication or deletion [10, 11]. From a mechanistic viewpoint, pre-transcriptional intragenic (gene body) CpG modification [12–14] by methylation writers [15] (methyltransferases) enables the site-specific DNA binding [16, 17] of trans-acting readers (methylcytosine-binding proteins, MBPs, such as MeCP2 or MBD2 [18–20])–germline dysfunctions of which may cause neurological disorders [21–23]–or else erasure by DNA methylation editors (TET proteins [24]). This epigenetic plasticity of 5-methylcytosine [25] (5mC; sometimes termed the fifth base of DNA [26]) has been proposed to be the basis of an alternate genomic code that diversifies gene function [27].

We have reported correlations between amino acid functionality and conservation of CpG-containing codons [28, 29] when compared with synonymous non-CpG codons such as may have arisen via methylation-dependent CG→TA transitions [30]. The importance of conserved exonic CpG sites is further implied by the biology of diseases like cancer in which mutations of such sites play a central role in pathogenesis [31]. It therefore seems likely that the letters of the genetic code contain meanings not limited to polypeptide synthesis. Yeast studies confirm that synonymous mutations yield deleterious phenotypes with similar efficiency to nonsynonymous mutations [32], reflecting DNA base-specific modifications of mRNA expression [33] or splicing [34].

*TP53*, a pivotal cell-cycle regulator [35–37], is the most mutated gene in human cancer [38]. CpG dinucleotides in exons 5–8 of this gene are constitutively methylated [39], predisposing to age- or carcinogen-induced mutations [40] that alter DNA binding by the encoded p53 proteins, and so dysregulate growth control [41, 42]. Missense mutations of this kind may not only abrogate tumor suppression, but also act in a dominant-negative manner to drive cancer progression [43]. Hence, as well as permitting carcinogenesis [44], CpG-related *TP53* mutations often confer behavioral aggressivity on tumors such as breast [45], colorectal [46], ovarian [47], prostate [48] and lung cancers [49].

Exons 5–8 of the human *TP53* and mouse *Trp53* genes encode the critical p53 DNA-binding domain [50]. These orthologous exons exhibit high (> 90%) sequence homology, including > 95% CpG site retention, and 100% conservation of the main cancer-predisposing hydrophilic *CG*X-encoded arginine residues at positions 158, 175, 248, 273 and 282 [51]. Perfect conservation of these codon-specific amino acids across mammalian, fish and amphibian species [52] supports the validity of using mouse synonymous *Trp53* knock-ins to infer human *TP53* functions, as implied by domain-swapped human p53 knock-in (Hupki) mice [53, 54], as well as by the tumorigenicity of murine *Trp53* mutations homologous to Li-Fraumeni-type *TP53* codon 175/273 mutations [55]. The strongly conserved germline CpG sites of human *TP53* are therefore fairly regarded as a hypermutable genomic Achilles heel for somatic cancer causation and progression (S1 Fig) [50].

We have shown in earlier work involving synonymous CpG-substituted human *TP53* cDNAs that abnormal demethylation of a *TP53* CpG site in exon 5 activates an intron 4 promoter, leading to production of a truncated protein isoform [56]. Such p53 isoforms are reported to exert anti-apoptotic effects that inhibit the cell-regulatory functions of full-length p53 [57–60], as could manifest as developmental malformations [61]. We have now used CRISPR/Cas9 to create knock-in mice expressing synonymous variable-CpG (vc) *Trp53* exons 5–8, and thus to test whether germline expression of one or both of these CpG extremes causes embryogenetic defects, impaired fertility, shortened survival, or altered susceptibility to spontaneous cancers, despite the predicted absence of change in the amino acid sequence of p53 proteins encoded.

## Materials and methods

### Synonymously substituted sequence design

In addition to knockout constructs (see below), three synonymous knock-in sequences were synthesised: (i) a putative ultra-stable (CpG-) exon 5–8 cDNA in which all 22 wild-type *Trp53* CpG sites were replaced by non-CpG dinucleotides predicted to encode the original amino acid sequence; (ii) a putative hyper-mutable (CpG+) cDNA in which existing CpG sites were retained, but a further 72 CpG sites were synonymously inserted; and (iii) a control non-CpG synonymous (NCpGS) mutated cDNA in which wild-type CpG sites remained unchanged, but 181 non-CpG sequences were altered in a manner predicted to leave the p53 amino acid structure intact. For the ultra-stable CpG- construct, all 22 CpG sites proved to be synonymously replaceable (S1 Table), re-emphasising the evolutionary question over their conservation.

For the hyper-mutable CpG+ construct, divergent CpG sites were substituted even when this did not reduce the number of sites–for example, ACG/GAG became ACC/GAG–to test further whether the precise location of a CpG base pairing contributes to its function. In addition, CpG adjacent sequences were changed if this could alter the evolutionarily selectable effects of spontaneous mutation; hence, the CGA trinucleotide was substituted by CGG, since CGA mutation to TGA causes a stop codon, whereas CGG to TGG specifies tryptophan (S2 Table).

### Knock-in mice

All *Trp53* mutant mouse lines were produced by the Mouse Engineering Garvan/ABR (MEGA) Facility (Moss Vale/Sydney, Australia) using CRISPR/Cas9 gene targeting in C57BL/6J mouse embryos using standard molecular and animal husbandry techniques [62, 63]. Two single guide RNAs (sgRNAs) targeted Cas9 cleavage 108 bp 5’ of exon 5 (ACCATTGGACGCCCTCG*CAGTGG) and 14 bp 3’ of exon 8 (GAGGTACGCAGGCGGGAG*CCAAGG) of *Trp53* (* = Cas9 cleavage site, underlined = proto-spacer-associated motif (PAM)). Three 3980 bp homologous recombination (HR) substrates were synthesized in pUC57 plasmid (Genscript, Piscataway, NJ), each of which included 1000 bp 5’ and 1500 bp 3’ homology arms either side of the two Cas9 target sequences and a G>C mutation in the 3’ base of each of the PAMs. The remainder of the substrates carried sequences corresponding to the intervening regions of *Trp53* gene but contained the specific base changes in exons 5–8 (S3–S5 Figs) designed to produce the CpG+, CpG- and NCpGS lines. In each case, a solution consisting of the two sgRNAs (15 ng/μl each), purified double stranded HR substrate plasmid DNA (2 ng/μl) and full length, polyadenylated *S*.*pyogenes* Cas9 mRNA (30 ng/μl) was prepared and microinjected into the nucleus and cytoplasm of C57BL/6J zygotes. Microinjected embryos were cultured overnight and those that underwent cleavage introduced into pseudo-pregnant foster mothers. Pups were screened by PCR to detect homologous recombination of each HR substrate into the *Trp53* alleles and founder animals crossed with C57BL/6J mice to establish the three *Trp53* knock-in lines. In addition, a mouse in which the sequences between the two Cas9 target sequences had been deleted (and hence, exons 5–8 removed) was used to establish a *Trp53* knockout line.

### Ethics and safety

Mice were bred at Australian BioResources (ABR; MossVale, NSW, Australia) and housed in specific pathogen-free conditions. All animal studies were approved and conducted in compliance with the guidelines set by the Garvan/St.Vincent’s Animal Ethics Committee. Mice in this study were monitored weekly for weight changes, signs of discomfort, or poor health; where humanely indicated by such endpoints (e.g., signs of pain, inability to reach food, or ≥ 20% weight loss), mice were sacrificed by carbon dioxide asphyxiation. No anaesthesia or analgesia was carried out. Pre-euthanasia causes of death are listed in Table 1. At any one time, 10–12 mice were analysed per genotype to ensure interpretability of observations.

**Table 1:** Observed phenotypes in transgenic mice.

Animal no.	Short line name	Genotype	Sex (M/F)	Survival (weeks)	Post mortem phenotype
115			M	22	Tumor
161			M	17	Tumor
317			M	28	Tumor
131		Homozygous *Trp53* Δ	M	19	Abdominal bloating
160			M	15	Facial abscess
318			M	16	Squashed
96	*Trp53* Δ		F	14	Dystocia
167			F	12	Dystocia
291		Heterozygous *Trp53* Δ	F	10	Dystocia
395			F	24	Dystocia
337			M	3	Hydrocephalus
332			F	30	Bite wounds
324			M	4	Unknown
4	*Trp53* (NCpGS)	Heterozygous *Trp53* (NCpGS)	F	22	Dystocia
65			F	18	Dystocia
116			F	19	Vaginal septum
182			F	18	Not documented
23			F	12	Malocclusion
38	*Trp53* (CpG-)	Heterozygous *Trp53* (CpG-)	F	3	Malocclusion
243			F	6	Malocclusion
133		Homozygous *Trp53* (CpG-)	M	6	Malocclusion
271	*Trp53* (CpG+)	Heterozygous *Trp53* (CpG+)	M	5	Malocclusion
199			M	5	Malocclusion
152		Homozygous *Trp53* (CpG+)	M	5	Malocclusion
117			M	3	Post-wean demise

Mice with *Trp53* knockout and/or CRISPR/*Cas9* knock-in using either vc-*Trp53* (CpG- or CpG+) or synonymous non-CpG *Trp53* (NCpGS) constructs were observed for developmental (e.g., congenital malformation) or adult (e.g., tumor) phenotypes.

## Results

Using the above approach we produced 1458 transgenic mice, comprising:

- 470 *Trp53*-null mice (from heterozygous knockout line ID 5555);
- 732 variable-CpG (vc)-*Trp53* mice, in turn comprising:
  ○ 322 *Trp53* CpG- mice, line ID 5801
  ○ 410 *Trp53* CpG+ mice, line ID 5774
- 256 synonymous non-CpG-mutated NCpGS *Trp53* mice, line ID 5775

The phenotypic observations from these knock-in mice are summarised in Table 1. As expected, tumor formation was evident in homozygous *Trp53* knockout mice (together with some other late-onset phenotypes suspicious for tumor growth, such as abdominal bloating and facial abscess, though the latter phenotype can also sometimes indicate subclinical incisor growth defects [64]; see below). In contrast, no tumors were detected in any subset (i.e., either CpG+ or CpG-) of vc-*Trp53* knock-in mice, nor in the NCpGS synonymous non-CpG mutation control. A high frequency of dystocia in *Trp53* homozygous knockout mice was also recorded; to the best of our knowledge, dystocia has not previously been reported as a feature of germline p53 loss of function, although it has been reported in double-knockout FasL-/p53- mice [65].

**Fig 1:**
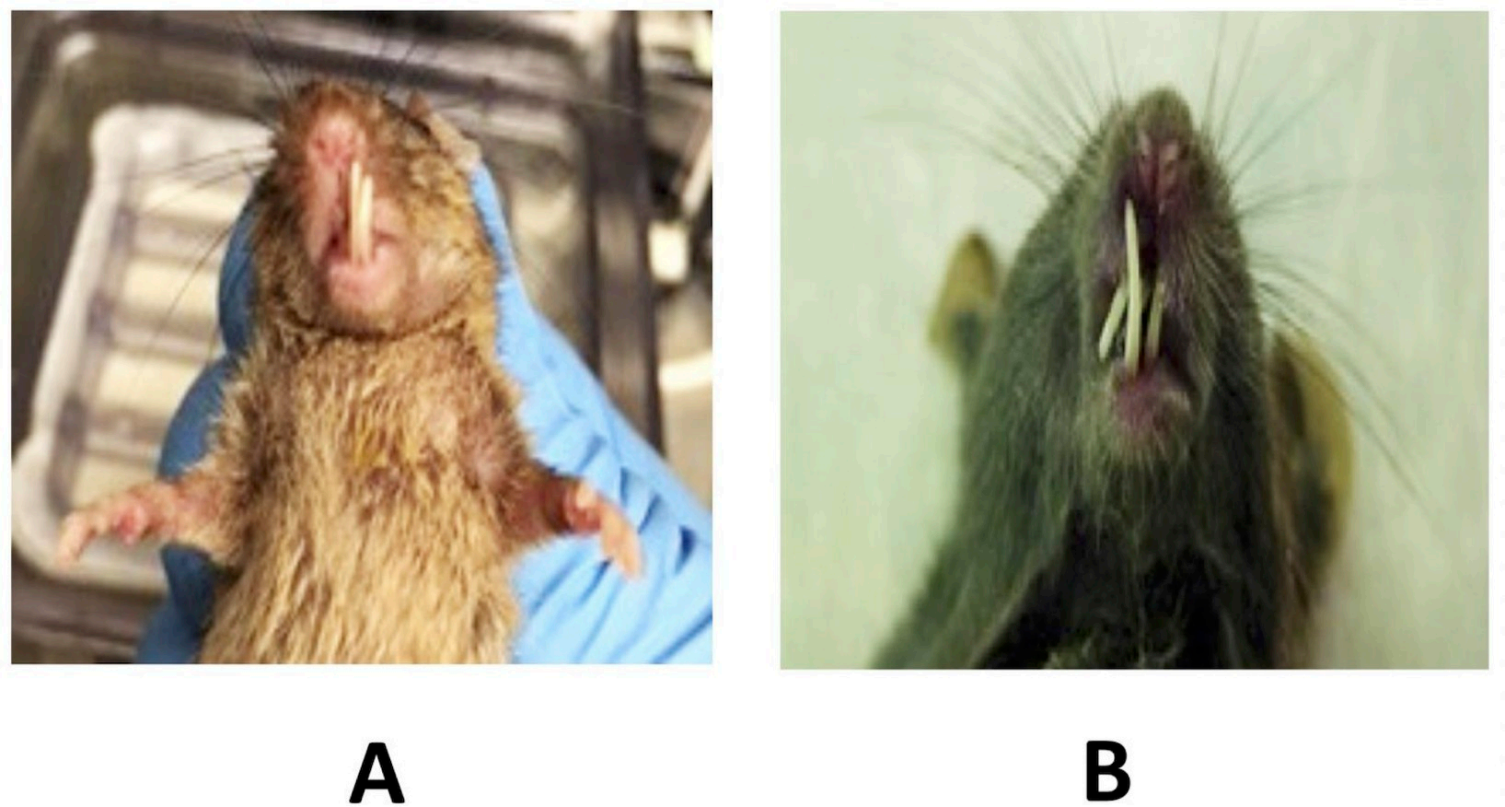
Typical examples of jaw malocclusion. A, Whole-mouse photograph showing the dysmorphic appearance of the incisors, as well as associated rapid malnutrition due to feeding difficulties. B, Detailed photo of incisor overgrowth defect underlying malocclusion and feeding problems.

Since the expected spontaneous frequency of malocclusion in C57BL/6J mice is 0.05%, i.e., one in 2000 [64], an unanticipated finding of this study was the clustering of six jaw malocclusion defects with vc-*Trp53* genotypes (Fig 1), compared to only one such malocclusion in non-vc-*Trp53* mice; conversely, vc-*Trp53* mice incurred only one other phenotype (labelled post-wean demise, with no other details), whereas non-vc-*Trp53* mice developed 17 other, non-malocclusion, phenotypes (χ^2^ with Yates correction = 12.3, p < .0001). Malocclusion was associated with premature mortality in vc-*Trp53* mice, with survival averaging only 5 +/- 0.4 weeks, presumably due to severe feeding problems (e.g., see Fig 1A); the average survival of other lethal phenotypes in this series was 17 +/- 1.7 weeks. The only non-vc-*Trp53* mouse with malocclusion (in the NCpGS genotype) survived for 12 weeks, i.e., longer than in the affected vc-*Trp53* mice.

A notable feature of the vc-*Trp53*-associated malocclusion cluster is that it was phenotypically evident with both CpG- and CpG+ vc-*Trp53 g*enotypes, as well as with heterozygous and homozygous gene dosages. This could suggest a dominant effect caused by aberrations that occur in the absence of evolutionarily conserved (i.e., correct) spatial CpG positioning, as discussed further below.

Beyond the above-noted effects on dental/jaw morphology, and the lack of any detectable effect on spontaneous tumor frequency, significant negatives of the study included no evident effect of vc-*Trp53* (or NCpGS) genotypes on mouse fertility or longevity.

## Discussion

Consistent with abnormal p53-dependent apoptosis, transgenic mice with p53 anomalies can develop upper incisor tooth malformations [66, 67]. Incisor defects likewise occur in rodent models of Rett syndrome, and lead to jaw malocclusion [68]; in this context, loss of normal MeCP2-dependent trans-repression [69] dysregulates mRNA splicing [70–72]. Relevant to this, the ability of wild-type MeCP2 to direct normal splicing events depends on exon recognition which is based in turn on correct spatial recruitment by intragenic methylation sites [73]. These observations are consistent with the hypothesis raised by ourselves and others that evolutionary CpG conservation could in part reflect a teratogenesis-mediated selection pressure which is operative in utero [74–76].

Hence, a plausible evo-devo view [77] of our results is that disrupting the conserved intragenic CpG structure of *Trp53* –in this study, either by synonymously removing all 22 exonic CpG sites, or else by inserting 72 synonymous CpG sites–deranges the topology of site-specific MBP (e.g., MeCP2 or MBD2) interactions with *Trp53*, leading to loss of pro-apoptotic gene function in the developing tooth bed [78, 79], e.g., by splice-dependent [80] or chromatin-based *trans*-repression mechanisms [81, 82]. Consistent with this, *TP53* is downregulated in proliferative dental pathologies such as odontogenic keratocysts and ameloblastomas [83]. Moreover, malregulation of the p53-interactive homolog p63 [67, 84, 85] is firmly implicated in tooth and craniofacial defects [86–88].

A question prompted by these findings relates to the fate of organisms with more extensive–e.g., genome-wide, rather than *Trp*53-specific–synonymous exonic variant-CpG genotypes. We speculate that such widespread CpG change could prove embryonic lethal, since it may cause intragenic dysregulatory effects on a scale equating to complete knockdown of all MBPs. Conversely, the negative finding of this study–viz., that no vc-*Trp53* knock-in mouse lineages developed spontaneous tumors, unlike their loss-of-function *Trp53* knockout controls–suggests that in vivo applications of this cellular approach, whether germline or somatic, may be safe.

It should be noted that there are additional explanations for these results which are independent of MBPs. For example, one possibility is that alterations of intragenic CpG sites could create or remove as-yet-unrecognised alternative transcription initiation sites that in turn alter function of the encoded protein [89, 90]. A second possibility is that gain or loss of CpG sites alters mRNA secondary structure and (hence) protein folding [91], perhaps via changes in base stacking and Z-RNA formation [92, 93] due to modification of CpG step number or position [94, 95]. Yet another possibility is that altered intragenic CpG content could affect ribosomal translational pausing with consequent downstream effects on protein translation efficiency or subdomain folding [96].

There remain several other important limitations of this study. The short-term mouse knock-in system is not a definitive in vivo test, as it does not exclude different results when assessed over other timescales or conditions [97]–such as over a human lifespan or, more crucially, as could relate to evolutionary CpG-dependent speciation events selected by environmental change [98–101]. Moreover, the finding that CpG-enriched and CpG-depleted constructs are both associated with malocclusion, as are homozygotes and heterozygotes, complicates interpretation; a qualitative explanation mandating a perfectly correct spatial arrangement of methyl-binding proteins is possible, and consistent with the tight evolutionary conservation of CpG sites, and the similar Rett phenotype. Finally, it remains unclear why this reported experimental phenotype is so restricted to orodental tissues, and how this might relate to a tissue-specific loss or change of p53 function, although intermediary effects on malocclusion-specific genes [102] or dental proteins [103, 104] are by no means excluded in the etiopathogenesis.

## Conclusion

The association between synonymous CpG-variant *Trp53* sequences and maldevelopment reported here supports, albeit indirectly, a growing body of epigenetic evidence favoring a CpG phenotype, though it is unclear whether this is due to pre-translational methylation-dependent interactions with DNA-binding trans-acting factors, or to MBP-unrelated changes in mRNA processing, structure, or translation. In other respects, the study suggests that synonymous replacement of conserved exonic CpG sites is well tolerated in unstressed mammalian tissues.

## Supporting information

S1 Fig Relationship between amino acid site-specificity of sporadic carcinogenic mutations (above) and evolutionary rate (Ka/Ks, below: red) in *TP53*. Sequences were downloaded from NCBI Entrez Gene (http://www.ncbi.nlm.nih.gov/Entrez/Gene), and homolog data in XML format from NCBI Homolo-Gene database (ftp://ftp.ncbi.nih.gov/pub/HomoloGene/). Mutation data were downloaded from the Human Gene Mutation Database. K-estimator 6.1 (with window size of 33 codons and step size of 10 codons using Kimura 2-parameter method) and PAML 3.15 with yn00 model were used for evolutionary rate calculations. Orthologous gene pairs between human and mouse, together with their synonymous substitution (Ks), nonsynonymous substitution rate (Ka), and their ratio (Ka/Ks), were thus isolated. The Ka/Ks evolutionary rate for *TP53* CpG sites in exons 5–8 was shown to approach zero, consistent with high negative selection pressure, with these same (functionally important) germline sites closely corresponding to those undergoing somatic mutation in tumors.
(TIF)

S2 Fig Illustration of mutagenesis strategy based on earlier in vitro studies using human *TP53* cDNA constructs. *A*, Representation of synonymous mutations introduced into cDNA constructs, exons 5–8. Open circles–ancestral CpG sites. Red symbols–additional synonymous CpG sites (CGN, NCG, NNC/GNN). The first (wild-type) cDNA lacks the three introns normally bridging exons 5–8; the second construct, with synonymous losses of wild-type CpG sites, is labeled stable; the third construct, to which synonymous CpG-containing sites have been added, is labeled missense. *B*, Hypothetical phenotypic effects as potential downstream somatic consequences of altered germline *TP53* mutation frequencies secondary to the synonymous changes.
(TIF)

S3 Fig Synonymous mutagenised *Trp53* CpG- sequence changes. 22 CpG sites highlighted in yellow are replaced as shown by synonymous green-highlighted bases.
(TIF)

S4 Fig Synonymous mutagenised *Trp53* CpG+ sequence changes. In addition to the retained wild-type CpG sites (highlighted in purple), 72 new CpG sites are created by synonymous replacement by the bases highlighted in either blue or yellow.
(TIF)

S5 Fig Mouse exons 5–8, all non-CpG-synonymous (NCpGS) versus WT. CpG sites highlighted in yellow remain conserved in both sequences. Green-highlighted bases in the NCpGS sequence represent all other (181) possible synonymous nucleotide changes.
(TIF)

S1 Table Exon-specific synonymous base changes in CpG- vc-*Trp53* mice. CpG sites are highlighted.
(TIF)

S2 Table Exon-specific synonymous base changes in CpG+ vc-*Trp53* mice. CpG sites are highlighted.
(TIF)

## References

[1] Minarovits J, Banati F, Szenthe K, Niller HH. Epigenetic Regulation. Adv Exp Med Biol. 2016;879:1–25. doi: 10.1007/978-3-319-24738-0_1 . 26659261

[2] Hernando-Herraez I, Garcia-Perez R, Sharp AJ, Marques-Bonet T. DNA Methylation: Insights into Human Evolution. PLoS Genet. 2015;11(12):e1005661. Epub 20151210. doi: 10.1371/journal.pgen.1005661 ; PubMed Central PMCID: PMC4684328. 26658498 PMC4684328

[3] Feng S, Cokus SJ, Zhang X, Chen PY, Bostick M, Goll MG, et al. Conservation and divergence of methylation patterning in plants and animals. Proc Natl Acad Sci U S A. 2010;107(19):8689–94. Epub 20100415. doi: 10.1073/pnas.1002720107 ; PubMed Central PMCID: PMC2889301. 20395551 PMC2889301

[4] Coll M, Solans X, Font-Altaba M, Subirana JA. Crystal and molecular structure of the sodium salt of the dinucleotide duplex d(CpG). J Biomol Struct Dyn. 1987;4(5):797–811. doi: 10.1080/07391102.1987.10507679 . 3270529

[5] Barone G, Fonseca Guerra C, Bickelhaupt FM. B-DNA Structure and Stability as Function of Nucleic Acid Composition: Dispersion-Corrected DFT Study of Dinucleoside Monophosphate Single and Double Strands. ChemistryOpen. 2013;2(5–6):186–93. Epub 20130816. doi: 10.1002/open.201300019 ; PubMed Central PMCID: PMC3892189. 24551565 PMC3892189

[6] Altun A, Garcia-Rates M, Neese F, Bistoni G. Unveiling the complex pattern of intermolecular interactions responsible for the stability of the DNA duplex. Chem Sci. 2021;12(38):12785–93. Epub 20210902. doi: 10.1039/d1sc03868k ; PubMed Central PMCID: PMC8494058. 34703565 PMC8494058

[7] Pfeifer GP. Mutagenesis at methylated CpG sequences. Curr Top Microbiol Immunol. 2006;301:259–81. doi: 10.1007/3-540-31390-7_10 . 16570852

[8] Rideout WM 3rd, Coetzee GA, Olumi AF, Jones PA. 5-Methylcytosine as an endogenous mutagen in the human LDL receptor and p53 genes. Science. 1990;249(4974):1288–90. Epub 1990/09/14. doi: 10.1126/science.1697983 . 1697983

[9] Tsunoyama K, Bellgard MI, Gojobori T. Intragenic variation of synonymous substitution rates is caused by nonrandom mutations at methylated CpG. J Mol Evol. 2001;53(4–5):456–64. doi: 10.1007/s002390010235 . 11675605

[10] Cooper DN, Krawczak M. Cytosine methylation and the fate of CpG dinucleotides in vertebrate genomes. Hum Genet. 1989;83(2):181–8. doi: 10.1007/BF00286715 . 2777259

[11] Kricker MC, Drake JW, Radman M. Duplication-targeted DNA methylation and mutagenesis in the evolution of eukaryotic chromosomes. Proc Natl Acad Sci U S A. 1992;89(3):1075–9. doi: 10.1073/pnas.89.3.1075 ; PubMed Central PMCID: PMC48388. 1736289 PMC48388

[12] Jjingo D, Conley AB, Yi SV, Lunyak VV, Jordan IK. On the presence and role of human gene-body DNA methylation. Oncotarget. 2012;3(4):462–74. doi: 10.18632/oncotarget.497 ; PubMed Central PMCID: PMC3380580. 22577155 PMC3380580

[13] Takuno S, Gaut BS. Body-methylated genes in Arabidopsis thaliana are functionally important and evolve slowly. Mol Biol Evol. 2012;29(1):219–27. Epub 20110802. doi: 10.1093/molbev/msr188 . 21813466

[14] Keller TE, Han P, Yi SV. Evolutionary Transition of Promoter and Gene Body DNA Methylation across Invertebrate-Vertebrate Boundary. Mol Biol Evol. 2016;33(4):1019–28. Epub 20151229. doi: 10.1093/molbev/msv345 ; PubMed Central PMCID: PMC4776710. 26715626 PMC4776710

[15] Jeltsch A, Broche J, Bashtrykov P. Molecular Processes Connecting DNA Methylation Patterns with DNA Methyltransferases and Histone Modifications in Mammalian Genomes. Genes (Basel). 2018;9(11). Epub 20181121. doi: 10.3390/genes9110566 ; PubMed Central PMCID: PMC6266221. 30469440 PMC6266221

[16] Rao S, Chiu TP, Kribelbauer JF, Mann RS, Bussemaker HJ, Rohs R. Systematic prediction of DNA shape changes due to CpG methylation explains epigenetic effects on protein-DNA binding. Epigenetics Chromatin. 2018;11(1):6. Epub 20180206. doi: 10.1186/s13072-018-0174-4 ; PubMed Central PMCID: PMC5800008. 29409522 PMC5800008

[17] Dantas Machado AC, Zhou T, Rao S, Goel P, Rastogi C, Lazarovici A, et al. Evolving insights on how cytosine methylation affects protein-DNA binding. Brief Funct Genomics. 2015;14(1):61–73. Epub 20141014. doi: 10.1093/bfgp/elu040 ; PubMed Central PMCID: PMC4303714. 25319759 PMC4303714

[18] Shimbo T, Wade PA. Proteins That Read DNA Methylation. Adv Exp Med Biol. 2016;945:303–20. doi: 10.1007/978-3-319-43624-1_13 . 27826844

[19] Du Q, Luu PL, Stirzaker C, Clark SJ. Methyl-CpG-binding domain proteins: readers of the epigenome. Epigenomics. 2015;7(6):1051–73. Epub 20150430. doi: 10.2217/epi.15.39 . 25927341

[20] Gunther K, Rust M, Leers J, Boettger T, Scharfe M, Jarek M, et al. Differential roles for MBD2 and MBD3 at methylated CpG islands, active promoters and binding to exon sequences. Nucleic Acids Res. 2013;41(5):3010–21. Epub 20130129. doi: 10.1093/nar/gkt035 ; PubMed Central PMCID: PMC3597697. 23361464 PMC3597697

[21] Guy J, Hendrich B, Holmes M, Martin JE, Bird A. A mouse Mecp2-null mutation causes neurological symptoms that mimic Rett syndrome. Nat Genet. 2001;27(3):322–6. doi: 10.1038/85899 . 11242117

[22] Weng SM, Bailey ME, Cobb SR. Rett syndrome: from bed to bench. Pediatr Neonatol. 2011;52(6):309–16. Epub 20111106. doi: 10.1016/j.pedneo.2011.08.002 . 22192257

[23] Hodge JC, Mitchell E, Pillalamarri V, Toler TL, Bartel F, Kearney HM, et al. Disruption of MBD5 contributes to a spectrum of psychopathology and neurodevelopmental abnormalities. Mol Psychiatry. 2014;19(3):368–79. Epub 20130416. doi: 10.1038/mp.2013.42 ; PubMed Central PMCID: PMC4756476. 23587880 PMC4756476

[24] Seethy A, Pethusamy K, Chattopadhyay I, Sah R, Chopra A, Dhar R, et al. TETology: Epigenetic Mastermind in Action. Appl Biochem Biotechnol. 2021;193(6):1701–26. Epub 20210310. doi: 10.1007/s12010-021-03537-5 . 33694104

[25] Hardwick JS, Lane AN, Brown T. Epigenetic Modifications of Cytosine: Biophysical Properties, Regulation, and Function in Mammalian DNA. Bioessays. 2018;40(3). Epub 20180125. doi: 10.1002/bies.201700199 . 29369386

[26] Lister R, Ecker JR. Finding the fifth base: genome-wide sequencing of cytosine methylation. Genome Res. 2009;19(6):959–66. Epub 20090309. doi: 10.1101/gr.083451.108 ; PubMed Central PMCID: PMC3807530. 19273618 PMC3807530

[27] Perez A, Castellazzi CL, Battistini F, Collinet K, Flores O, Deniz O, et al. Impact of methylation on the physical properties of DNA. Biophys J. 2012;102(9):2140–8. doi: 10.1016/j.bpj.2012.03.056 ; PubMed Central PMCID: PMC3341543. 22824278 PMC3341543

[28] Epstein RJ, Lin K, Tan TW. A functional significance for codon third bases. Gene. 2000;245(2):291–8. doi: 10.1016/s0378-1119(00)00042-1 . 10717480

[29] Lin K, Tan SB, Kolatkar PR, Epstein RJ. Nonrandom intragenic variations in patterns of codon bias implicate a sequential interplay between transitional genetic drift and functional amino acid selection. J Mol Evol. 2003;57(5):538–45. doi: 10.1007/s00239-003-2507-5 . 14738312

[30] Klutstein M, Nejman D, Greenfield R, Cedar H. DNA Methylation in Cancer and Aging. Cancer Res. 2016;76(12):3446–50. Epub 20160602. doi: 10.1158/0008-5472.CAN-15-3278 . 27256564

[31] Yoon JH, Smith LE, Feng Z, Tang M, Lee CS, Pfeifer GP. Methylated CpG dinucleotides are the preferential targets for G-to-T transversion mutations induced by benzo [a]pyrene diol epoxide in mammalian cells: similarities with the p53 mutation spectrum in smoking-associated lung cancers. Cancer Res. 2001;61(19):7110–7. . 11585742

[32] Shen X, Song S, Li C, Zhang J. Synonymous mutations in representative yeast genes are mostly strongly non-neutral. Nature. 2022. Epub 20220608. doi: 10.1038/s41586-022-04823-w . 35676473 PMC9650438

[33] Sharp N. Mutations matter even if proteins stay the same. Nature. 2022. Epub 20220608. doi: 10.1038/d41586-022-01091-6 . 35676345

[34] Bhagavatula G, Rich MS, Young DL, Marin M, Fields S. A Massively Parallel Fluorescence Assay to Characterize the Effects of Synonymous Mutations on TP53 Expression. Mol Cancer Res. 2017;15(10):1301–7. Epub 20170626. doi: 10.1158/1541-7786.MCR-17-0245 ; PubMed Central PMCID: PMC5626615. 28652265 PMC5626615

[35] Manfredi JJ. p53 and Development: Shedding Light on an Evolutionary Enigma. Dev Cell. 2019;50(2):128–9. doi: 10.1016/j.devcel.2019.07.004 . 31336095

[36] Boutelle AM, Attardi LD. p53 and Tumor Suppression: It Takes a Network. Trends Cell Biol. 2021;31(4):298–310. Epub 20210128. doi: 10.1016/j.tcb.2020.12.011 ; PubMed Central PMCID: PMC7954925. 33518400 PMC7954925

[37] Hollstein M, Hainaut P. Massively regulated genes: the example of TP53. J Pathol. 2010;220(2):164–73. doi: 10.1002/path.2637 . 19918835

[38] Joerger AC, Fersht AR. Structural biology of the tumor suppressor p53 and cancer-associated mutants. Adv Cancer Res. 2007;97:1–23. doi: 10.1016/S0065-230X(06)97001-8 . 17419939

[39] Tornaletti S, Pfeifer GP. Complete and tissue-independent methylation of CpG sites in the p53 gene: implications for mutations in human cancers. Oncogene. 1995;10(8):1493–9. . 7731703

[40] Magewu AN, Jones PA. Ubiquitous and tenacious methylation of the CpG site in codon 248 of the p53 gene may explain its frequent appearance as a mutational hot spot in human cancer. Mol Cell Biol. 1994;14(6):4225–32. Epub 1994/06/01. doi: 10.1128/mcb.14.6.4225-4232.1994 ; PubMed Central PMCID: PMC358788. 8196660 PMC358788

[41] Sigal A, Rotter V. Oncogenic mutations of the p53 tumor suppressor: the demons of the guardian of the genome. Cancer Res. 2000;60(24):6788–93. . 11156366

[42] Song H, Xu Y. Gain of function of p53 cancer mutants in disrupting critical DNA damage response pathways. Cell Cycle. 2007;6(13):1570–3. Epub 20070522. doi: 10.4161/cc.6.13.4456 . 17598983

[43] Wang Y, Suh YA, Fuller MY, Jackson JG, Xiong S, Terzian T, et al. Restoring expression of wild-type p53 suppresses tumor growth but does not cause tumor regression in mice with a p53 missense mutation. J Clin Invest. 2011;121(3):893–904. doi: 10.1172/JCI44504 ; PubMed Central PMCID: PMC3049366. 21285512 PMC3049366

[44] Zhao Y, Epstein RJ. Programmed genetic instability: a tumor-permissive mechanism for maintaining the evolvability of higher species through methylation-dependent mutation of DNA repair genes in the male germ line. Mol Biol Evol. 2008;25(8):1737–49. Epub 20080604. doi: 10.1093/molbev/msn126 ; PubMed Central PMCID: PMC2464741. 18535014 PMC2464741

[45] Petitjean A, Achatz MI, Borresen-Dale AL, Hainaut P, Olivier M. TP53 mutations in human cancers: functional selection and impact on cancer prognosis and outcomes. Oncogene. 2007;26(15):2157–65. doi: 10.1038/sj.onc.1210302 . 17401424

[46] Iacopetta B, Russo A, Bazan V, Dardanoni G, Gebbia N, Soussi T, et al. Functional categories of TP53 mutation in colorectal cancer: results of an International Collaborative Study. Ann Oncol. 2006;17(5):842–7. Epub 20060308. doi: 10.1093/annonc/mdl035 . 16524972

[47] Kalamanathan S, Bates V, Lord R, Green JA. The mutational profile of sporadic epithelial ovarian carcinoma. Anticancer Res. 2011;31(8):2661–8. . 21778320

[48] Schlomm T, Iwers L, Kirstein P, Jessen B, Kollermann J, Minner S, et al. Clinical significance of p53 alterations in surgically treated prostate cancers. Mod Pathol. 2008;21(11):1371–8. Epub 20080613. doi: 10.1038/modpathol.2008.104 . 18552821

[49] Campling BG, El-Deiry WS. Clinical implication of p53 mutation in lung cancer. Mol Biotechnol. 2003;24(2):141–56. doi: 10.1385/MB:24:2:141 . 12746555

[50] Baugh EH, Ke H, Levine AJ, Bonneau RA, Chan CS. Why are there hotspot mutations in the TP53 gene in human cancers? Cell Death Differ. 2018;25(1):154–60. Epub 20171103. doi: 10.1038/cdd.2017.180 ; PubMed Central PMCID: PMC5729536. 29099487 PMC5729536

[51] Zakut-Houri R, Bienz-Tadmor B, Givol D, Oren M. Human p53 cellular tumor antigen: cDNA sequence and expression in COS cells. EMBO J. 1985;4(5):1251–5. doi: 10.1002/j.1460-2075.1985.tb03768.x ; PubMed Central PMCID: PMC554332. 4006916 PMC554332

[52] Hollstein M, Sidransky D, Vogelstein B, Harris CC. p53 mutations in human cancers. Science. 1991;253(5015):49–53. doi: 10.1126/science.1905840 . 1905840

[53] Luo JL, Yang Q, Tong WM, Hergenhahn M, Wang ZQ, Hollstein M. Knock-in mice with a chimeric human/murine p53 gene develop normally and show wild-type p53 responses to DNA damaging agents: a new biomedical research tool. Oncogene. 2001;20(3):320–8. doi: 10.1038/sj.onc.1204080 . 11313961

[54] Kim SI, Hollstein M, Pfeifer GP, Besaratinia A. Unveiling the methylation status of CpG dinucleotides in the substituted segment of the human p53 knock-in (Hupki) mouse genome. Mol Carcinog. 2010;49(12):999–1006. Epub 2010/10/15. doi: 10.1002/mc.20683 ; PubMed Central PMCID: PMC2991417. 20945503 PMC2991417

[55] Olive KP, Tuveson DA, Ruhe ZC, Yin B, Willis NA, Bronson RT, et al. Mutant p53 gain of function in two mouse models of Li-Fraumeni syndrome. Cell. 2004;119(6):847–60. doi: 10.1016/j.cell.2004.11.004 . 15607980

[56] Blackburn J, Roden DL, Ng R, Wu J, Bosman A, Epstein RJ. Damage-inducible intragenic demethylation of the human TP53 tumor suppressor gene is associated with transcription from an alternative intronic promoter. Mol Carcinog. 2016;55(12):1940–51. Epub 20151216. doi: 10.1002/mc.22441 ; PubMed Central PMCID: PMC5111752. 26676339 PMC5111752

[57] Chen J, Ng SM, Chang C, Zhang Z, Bourdon JC, Lane DP, et al. p53 isoform delta113p53 is a p53 target gene that antagonizes p53 apoptotic activity via BclxL activation in zebrafish. Genes Dev. 2009;23(3):278–90. doi: 10.1101/gad.1761609 ; PubMed Central PMCID: PMC2648546. 19204115 PMC2648546

[58] Gong L, Gong H, Pan X, Chang C, Ou Z, Ye S, et al. p53 isoform Delta113p53/Delta133p53 promotes DNA double-strand break repair to protect cell from death and senescence in response to DNA damage. Cell Res. 2015;25(3):351–69. Epub 20150220. doi: 10.1038/cr.2015.22 ; PubMed Central PMCID: PMC4349251. 25698579 PMC4349251

[59] Shi H, Tao T, Huang D, Ou Z, Chen J, Peng J. A naturally occurring 4-bp deletion in the intron 4 of p53 creates a spectrum of novel p53 isoforms with anti-apoptosis function. Nucleic Acids Res. 2015;43(2):1035–43. Epub 20141229. doi: 10.1093/nar/gku1359 ; PubMed Central PMCID: PMC4333405. 25550427 PMC4333405

[60] Courtois S, Verhaegh G, North S, Luciani MG, Lassus P, Hibner U, et al. DeltaN-p53, a natural isoform of p53 lacking the first transactivation domain, counteracts growth suppression by wild-type p53. Oncogene. 2002;21(44):6722–8. doi: 10.1038/sj.onc.1205874 . 12360399

[61] Joruiz SM, Bourdon JC. p53 Isoforms: Key Regulators of the Cell Fate Decision. Cold Spring Harb Perspect Med. 2016;6(8). Epub 20160801. doi: 10.1101/cshperspect.a026039 ; PubMed Central PMCID: PMC4968168. 26801896 PMC4968168

[62] Yang H, Wang H, Jaenisch R. Generating genetically modified mice using CRISPR/Cas-mediated genome engineering. Nat Protoc. 2014;9(8):1956–68. Epub 20140724. doi: 10.1038/nprot.2014.134 . 25058643

[63] Platt RJ, Chen S, Zhou Y, Yim MJ, Swiech L, Kempton HR, et al. CRISPR-Cas9 knockin mice for genome editing and cancer modeling. Cell. 2014;159(2):440–55. Epub 20140925. doi: 10.1016/j.cell.2014.09.014 ; PubMed Central PMCID: PMC4265475. 25263330 PMC4265475

[64] Garcia-Arocena D. How to spot and manage malocclusion in research mice: The Jackson Laboratories; 2016. Available from: https://www.jax.org/news-and-insights/jax-blog/2016/july/how-to-spot-malocclusion-in-research-mice.

[65] Embree-Ku M, Boekelheide K. Absence of p53 and FasL has sexually dimorphic effects on both development and reproduction. Exp Biol Med (Maywood). 2002;227(7):545–53. doi: 10.1177/153537020222700720 . 12094020

[66] Armstrong JF, Kaufman MH, Harrison DJ, Clarke AR. High-frequency developmental abnormalities in p53-deficient mice. Curr Biol. 1995;5(8):931–6. doi: 10.1016/s0960-9822(95)00183-7 . 7583151

[67] Murray-Zmijewski F, Lane DP, Bourdon JC. p53/p63/p73 isoforms: an orchestra of isoforms to harmonise cell differentiation and response to stress. Cell Death Differ. 2006;13(6):962–72. doi: 10.1038/sj.cdd.4401914 . 16601753

[68] Wu Y, Zhong W, Cui N, Johnson CM, Xing H, Zhang S, et al. Characterization of Rett Syndrome-like phenotypes in Mecp2-knockout rats. J Neurodev Disord. 2016;8:23. Epub 20160616. doi: 10.1186/s11689-016-9156-7 ; PubMed Central PMCID: PMC4910223. 27313794 PMC4910223

[69] Lu S, Chen Y, Wang Z. Advances in the pathogenesis of Rett syndrome using cell models. Animal Model Exp Med. 2022. Epub 20220704. doi: 10.1002/ame2.12236 . 35785421 PMC9773312

[70] Li R, Dong Q, Yuan X, Zeng X, Gao Y, Chiao C, et al. Misregulation of Alternative Splicing in a Mouse Model of Rett Syndrome. PLoS Genet. 2016;12(6):e1006129. Epub 20160628. doi: 10.1371/journal.pgen.1006129 ; PubMed Central PMCID: PMC4924826. 27352031 PMC4924826

[71] Osenberg S, Karten A, Sun J, Li J, Charkowick S, Felice CA, et al. Activity-dependent aberrations in gene expression and alternative splicing in a mouse model of Rett syndrome. Proc Natl Acad Sci U S A. 2018;115(23):E5363–E72. Epub 20180516. doi: 10.1073/pnas.1722546115 ; PubMed Central PMCID: PMC6003366. 29769330 PMC6003366

[72] Young JI, Hong EP, Castle JC, Crespo-Barreto J, Bowman AB, Rose MF, et al. Regulation of RNA splicing by the methylation-dependent transcriptional repressor methyl-CpG binding protein 2. Proc Natl Acad Sci U S A. 2005;102(49):17551–8. Epub 20051026. doi: 10.1073/pnas.0507856102 ; PubMed Central PMCID: PMC1266160. 16251272 PMC1266160

[73] Maunakea AK, Nagarajan RP, Bilenky M, Ballinger TJ, D’Souza C, Fouse SD, et al. Conserved role of intragenic DNA methylation in regulating alternative promoters. Nature. 2010;466(7303):253–7. doi: 10.1038/nature09165 ; PubMed Central PMCID: PMC3998662. 20613842 PMC3998662

[74] Zhao Y, Epstein RJ. Conserved nonsense-prone CpG sites in apoptosis-regulatory tumour suppressor genes: stop signs on the road to cell death. J Evol Bioinformatics. 2013:(in press). Epub 2013. 10.4137/EBO.S11759 PMC3728200 23908585

[75] Torchinsky A, Toder V. Mechanisms of the embryo’s response to embryopathic stressors: a focus on p53. J Reprod Immunol. 2010;85(1):76–80. Epub 20100312. doi: 10.1016/j.jri.2010.01.003 . 20227113

[76] Gueta K, Molotski N, Gerchikov N, Mor E, Savion S, Fein A, et al. Teratogen-induced alterations in microRNA-34, microRNA-125b and microRNA-155 expression: correlation with embryonic p53 genotype and limb phenotype. BMC Dev Biol. 2010;10:20. Epub 20100221. doi: 10.1186/1471-213X-10-20 ; PubMed Central PMCID: PMC2841584. 20170545 PMC2841584

[77] Parsons KJ. Conservation biology meets evo-devo: How understanding the emergence of variation can inform its management. Evol Dev. 2021;23(4):269–72. doi: 10.1111/ede.12389 . 34478229

[78] Muica Nagy-Bota MC, Pap Z, Denes L, Ghizdavat A, Brinzaniuc K, Lup Cosarca AS, et al. Immunohistochemical study of Ki67, CD34 and p53 expression in human tooth buds. Rom J Morphol Embryol. 2014;55(1):43–8. . 24715164

[79] Yang X, Zhou Z, Mao Z, Shen M, Chen N, Miao D. Role of p53 deficiency in socket healing after tooth extractions. J Mol Histol. 2020;51(1):55–65. Epub 20200131. doi: 10.1007/s10735-020-09856-x . 32006186

[80] Lev Maor G, Yearim A, Ast G. The alternative role of DNA methylation in splicing regulation. Trends Genet. 2015;31(5):274–80. Epub 20150330. doi: 10.1016/j.tig.2015.03.002 . 25837375

[81] Nakao M, Matsui S, Yamamoto S, Okumura K, Shirakawa M, Fujita N. Regulation of transcription and chromatin by methyl-CpG binding protein MBD1. Brain Dev. 2001;23 Suppl 1:S174-6. doi: 10.1016/s0387-7604(01)00348-5 . 11738867

[82] Kinde B, Wu DY, Greenberg ME, Gabel HW. DNA methylation in the gene body influences MeCP2-mediated gene repression. Proc Natl Acad Sci U S A. 2016;113(52):15114–9. Epub 20161213. doi: 10.1073/pnas.1618737114 ; PubMed Central PMCID: PMC5206576. 27965390 PMC5206576

[83] Ghafouri-Fard S, Atarbashi-Moghadam S, Taheri M. Genetic factors in the pathogenesis of ameloblastoma, dentigerous cyst and odontogenic keratocyst. Gene. 2021;771:145369. Epub 20201217. doi: 10.1016/j.gene.2020.145369 . 33346102

[84] Levrero M, De Laurenzi V, Costanzo A, Gong J, Wang JY, Melino G. The p53/p63/p73 family of transcription factors: overlapping and distinct functions. J Cell Sci. 2000;113 (Pt 10):1661–70. doi: 10.1242/jcs.113.10.1661 . 10769197

[85] Osada M, Park HL, Nagakawa Y, Yamashita K, Fomenkov A, Kim MS, et al. Differential recognition of response elements determines target gene specificity for p53 and p63. Mol Cell Biol. 2005;25(14):6077–89. doi: 10.1128/MCB.25.14.6077-6089.2005 ; PubMed Central PMCID: PMC1168821. 15988020 PMC1168821

[86] Rufini A, Barlattani A, Docimo R, Velletri T, Niklison-Chirou MV, Agostini M, et al. p63 in tooth development. Biochem Pharmacol. 2011;82(10):1256–61. Epub 20110720. doi: 10.1016/j.bcp.2011.07.068 . 21787761

[87] Raj MT, Boughner JC. Detangling the evolutionary developmental integration of dentate jaws: evidence that a p63 gene network regulates odontogenesis exclusive of mandible morphogenesis. Evol Dev. 2016;18(5–6):317–23. doi: 10.1111/ede.12208 . 27870215

[88] Gupta R, Chaudhary M, Patil S, Fating C, Hande A, Suryawanshi H. Expression of p63 in tooth germ, dentigerous cyst and ameloblastoma. J Oral Maxillofac Pathol. 2019;23(1):43–8. doi: 10.4103/jomfp.JOMFP_125_18 ; PubMed Central PMCID: PMC6503805. 31110415 PMC6503805

[89] Neri F, Rapelli S, Krepelova A, Incarnato D, Parlato C, Basile G, et al. Intragenic DNA methylation prevents spurious transcription initiation. Nature. 2017;543(7643):72–7. Epub 20170222. doi: 10.1038/nature21373 . 28225755

[90] Neininger K, Marschall T, Helms V. SNP and indel frequencies at transcription start sites and at canonical and alternative translation initiation sites in the human genome. PLoS One. 2019;14(4):e0214816. Epub 20190412. doi: 10.1371/journal.pone.0214816 ; PubMed Central PMCID: PMC6461226. 30978217 PMC6461226

[91] Faure G, Ogurtsov AY, Shabalina SA, Koonin EV. Role of mRNA structure in the control of protein folding. Nucleic Acids Res. 2016;44(22):10898–911. Epub 20160727. doi: 10.1093/nar/gkw671 ; PubMed Central PMCID: PMC5159526. 27466388 PMC5159526

[92] Uesugi S, Shida T, Ikehara M. Synthesis and properties of CpG analogues containing an 8-bromoguanosine residue. Evidence for Z-RNA duplex formation. Biochemistry. 1982;21(14):3400–8. doi: 10.1021/bi00257a024 . 7115677

[93] Nichols PJ, Bevers S, Henen M, Kieft JS, Vicens Q, Vogeli B. Recognition of non-CpG repeats in Alu and ribosomal RNAs by the Z-RNA binding domain of ADAR1 induces A-Z junctions. Nat Commun. 2021;12(1):793. Epub 20210204. doi: 10.1038/s41467-021-21039-0 ; PubMed Central PMCID: PMC7862695. 33542240 PMC7862695

[94] Popenda M, Milecki J, Adamiak RW. High salt solution structure of a left-handed RNA double helix. Nucleic Acids Res. 2004;32(13):4044–54. Epub 20040803. doi: 10.1093/nar/gkh736 ; PubMed Central PMCID: PMC506817. 15292450 PMC506817

[95] D’Ascenzo L, Leonarski F, Vicens Q, Auffinger P. ’Z-DNA like’ fragments in RNA: a recurring structural motif with implications for folding, RNA/protein recognition and immune response. Nucleic Acids Res. 2016;44(12):5944–56. Epub 20160505. doi: 10.1093/nar/gkw388 ; PubMed Central PMCID: PMC4937326. 27151194 PMC4937326

[96] Postnikova OA, Uppal S, Huang W, Kane MA, Villasmil R, Rogozin IB, et al. The Functional Consequences of the Novel Ribosomal Pausing Site in SARS-CoV-2 Spike Glycoprotein RNA. Int J Mol Sci. 2021;22(12). Epub 20210617. doi: 10.3390/ijms22126490 ; PubMed Central PMCID: PMC8235447. 34204305 PMC8235447

[97] Acar M, Mettetal JT, van Oudenaarden A. Stochastic switching as a survival strategy in fluctuating environments. Nat Genet. 2008;40(4):471–5. Epub 2008/03/26. doi: 10.1038/ng.110 [pii] . 18362885

[98] Zemojtel T, Kielbasa SM, Arndt PF, Behrens S, Bourque G, Vingron M. CpG deamination creates transcription factor-binding sites with high efficiency. Genome Biol Evol. 2011;3:1304–11. Epub 20111019. doi: 10.1093/gbe/evr107 ; PubMed Central PMCID: PMC3228489. 22016335 PMC3228489

[99] Pertille F, Da Silva VH, Johansson AM, Lindstrom T, Wright D, Coutinho LL, et al. Mutation dynamics of CpG dinucleotides during a recent event of vertebrate diversification. Epigenetics. 2019;14(7):685–707. Epub 20190509. doi: 10.1080/15592294.2019.1609868 ; PubMed Central PMCID: PMC6557589. 31070073 PMC6557589

[100] Storz JF, Natarajan C, Signore AV, Witt CC, McCandlish DM, Stoltzfus A. The role of mutation bias in adaptive molecular evolution: insights from convergent changes in protein function. Philos Trans R Soc Lond B Biol Sci. 2019;374(1777):20180238. Epub 20190603. doi: 10.1098/rstb.2018.0238 ; PubMed Central PMCID: PMC6560279. 31154983 PMC6560279

[101] Chuang TJ, Chen FC, Chen YZ. Position-dependent correlations between DNA methylation and the evolutionary rates of mammalian coding exons. Proc Natl Acad Sci U S A. 2012;109(39):15841–6. Epub 20120910. doi: 10.1073/pnas.1208214109 ; PubMed Central PMCID: PMC3465446. 23019368 PMC3465446

[102] Boily G, He XH, Jardine K, McBurney MW. Disruption of Igfbp1 fails to rescue the phenotype of Sirt1-/- mice. Exp Cell Res. 2010;316(13):2189–93. Epub 20100420. doi: 10.1016/j.yexcr.2010.04.012 . 20412791

[103] Yamazaki H, Kunisada T, Miyamoto A, Tagaya H, Hayashi S. Tooth-specific expression conferred by the regulatory sequences of rat dentin sialoprotein gene in transgenic mice. Biochem Biophys Res Commun. 1999;260(2):433–40. doi: 10.1006/bbrc.1999.0875 . 10403786

[104] He B, Chiba Y, Li H, de Vega S, Tanaka K, Yoshizaki K, et al. Identification of the Novel Tooth-Specific Transcription Factor AmeloD. J Dent Res. 2019;98(2):234–41. Epub 20181114. doi: 10.1177/0022034518808254 ; PubMed Central PMCID: PMC6761735. 30426815 PMC6761735

