# Supplementary figures and images for: Synonymous alterations of cancer-associated Trp53 CpG mutational hotspots cause fatal developmental jaw malocclusions but no tumors in knock-in mice
Source: PLoS One. 2023 Apr 13;18(4):e0284327. doi: 10.1371/journal.pone.0284327 (PMC10101519; doi:10.1371/journal.pone.0284327)

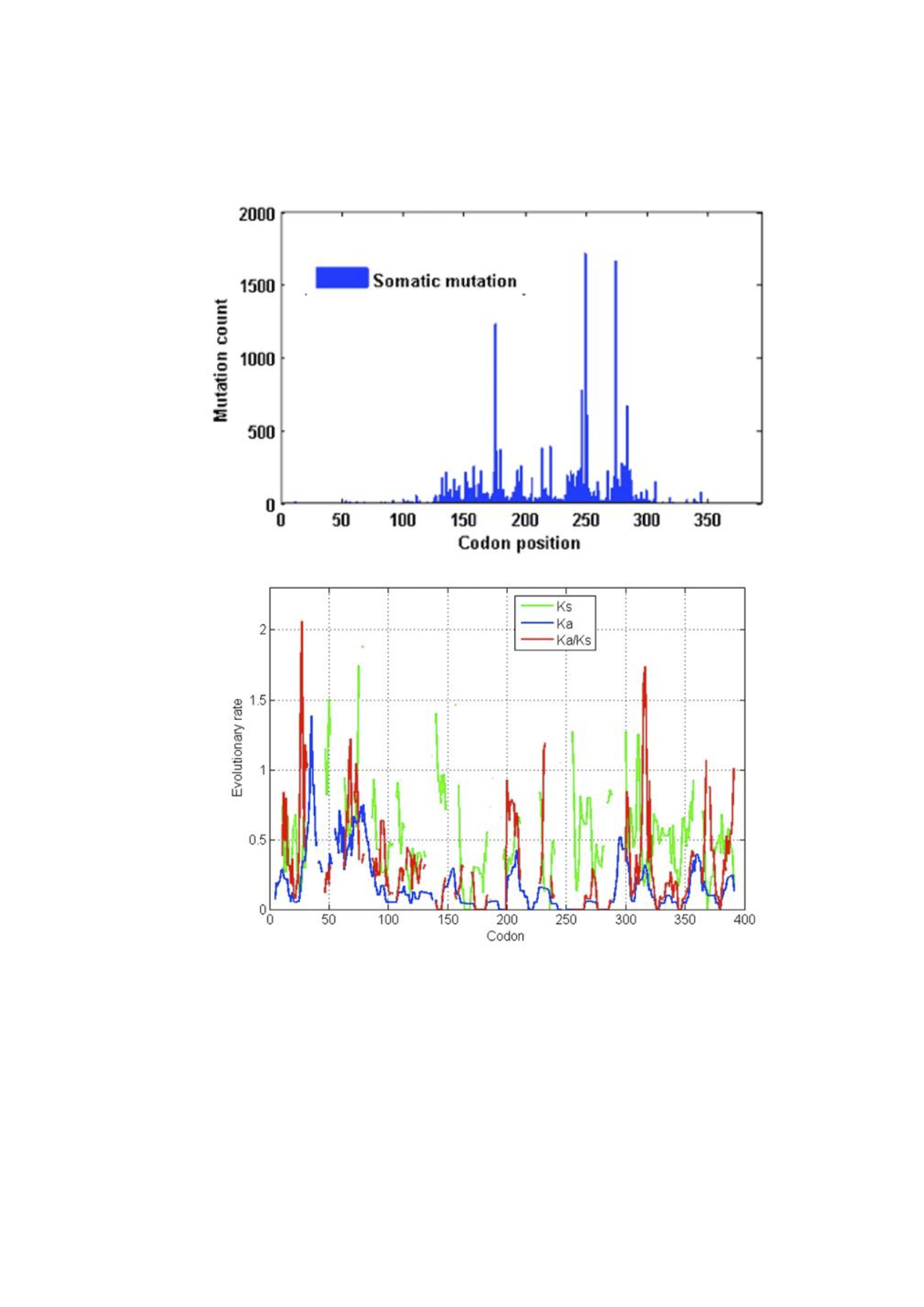

Supplement: S1 Fig — Sequences were downloaded from NCBI Entrez Gene (http://www.ncbi.nlm.nih.gov/Entrez/Gene), and homolog data in XML format from NCBI Homolo-Gene database (ftp://ftp.ncbi.nih.gov/pub/HomoloGene/). Mutation data were downloaded from the Human Gene Mutation Database. K-estimator 6.1 (with window size of 33 codons and step size of 10 codons using Kimura 2-parameter method) and PAML 3.15 with yn00 model were used for evolutionary rate calculations. Orthologous gene pairs between human and mouse, together with their synonymous substitution (Ks), nonsynonymous substitution rate (Ka), and their ratio (Ka/Ks), were thus isolated. The Ka/Ks evolutionary rate for TP53 CpG sites in exons 5–8 was shown to approach zero, consistent with high negative selection pressure, with these same (functionally important) germline sites closely corresponding to those undergoing somatic mutation in tumors. (TIF) [file pone.0284327.s001.tif]

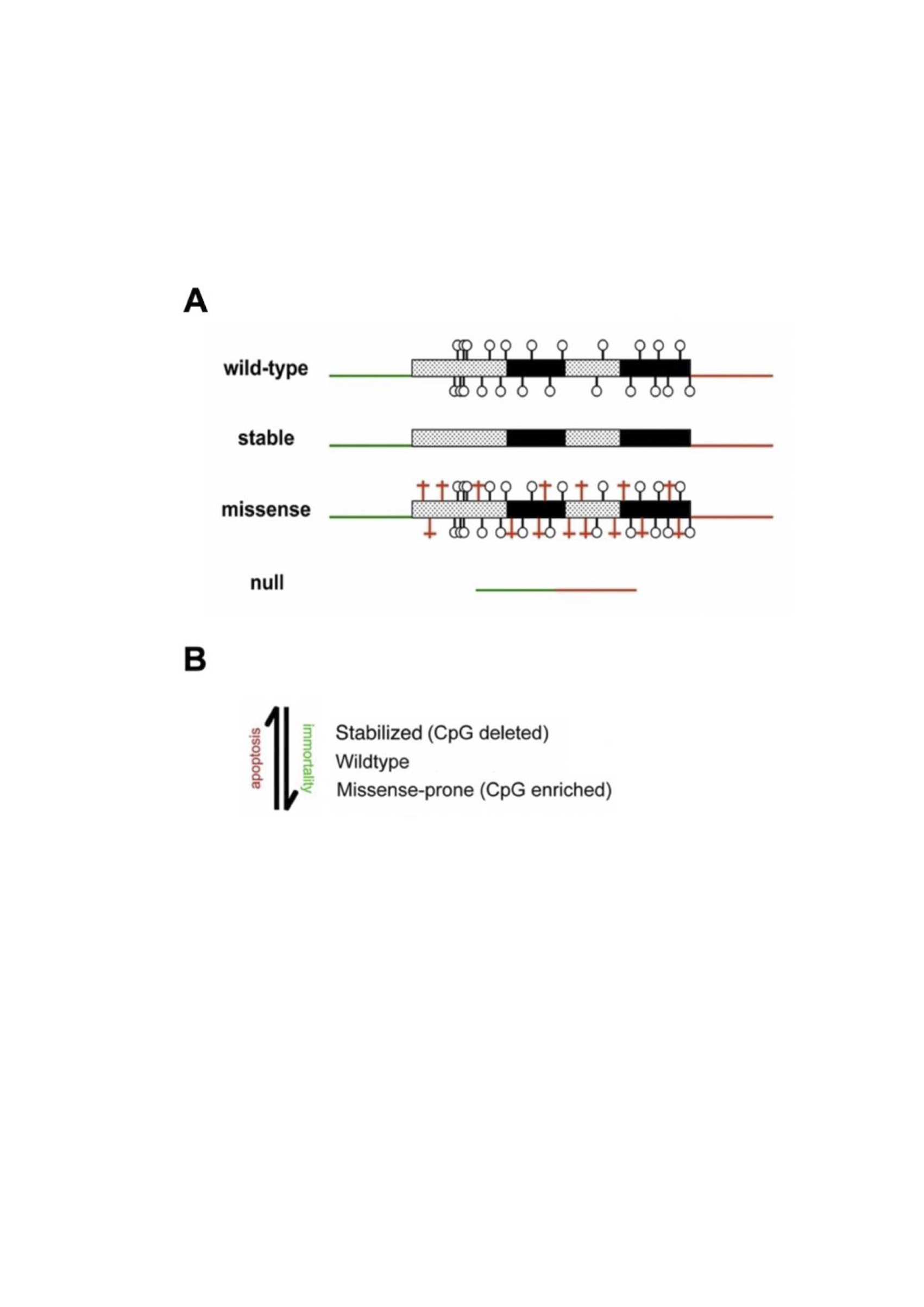

Supplement: S2 Fig — A, Representation of synonymous mutations introduced into cDNA constructs, exons 5–8. Open circles–ancestral CpG sites. Red symbols–additional synonymous CpG sites (CGN, NCG, NNC/GNN). The first (wild-type) cDNA lacks the three introns normally bridging exons 5–8; the second construct, with synonymous losses of wild-type CpG sites, is labeled stable; the third construct, to which synonymous CpG-containing sites have been added, is labeled missense. B, Hypothetical phenotypic effects as potential downstream somatic consequences of altered germline TP53 mutation frequencies secondary to the synonymous changes. (TIF) [file pone.0284327.s002.tif]

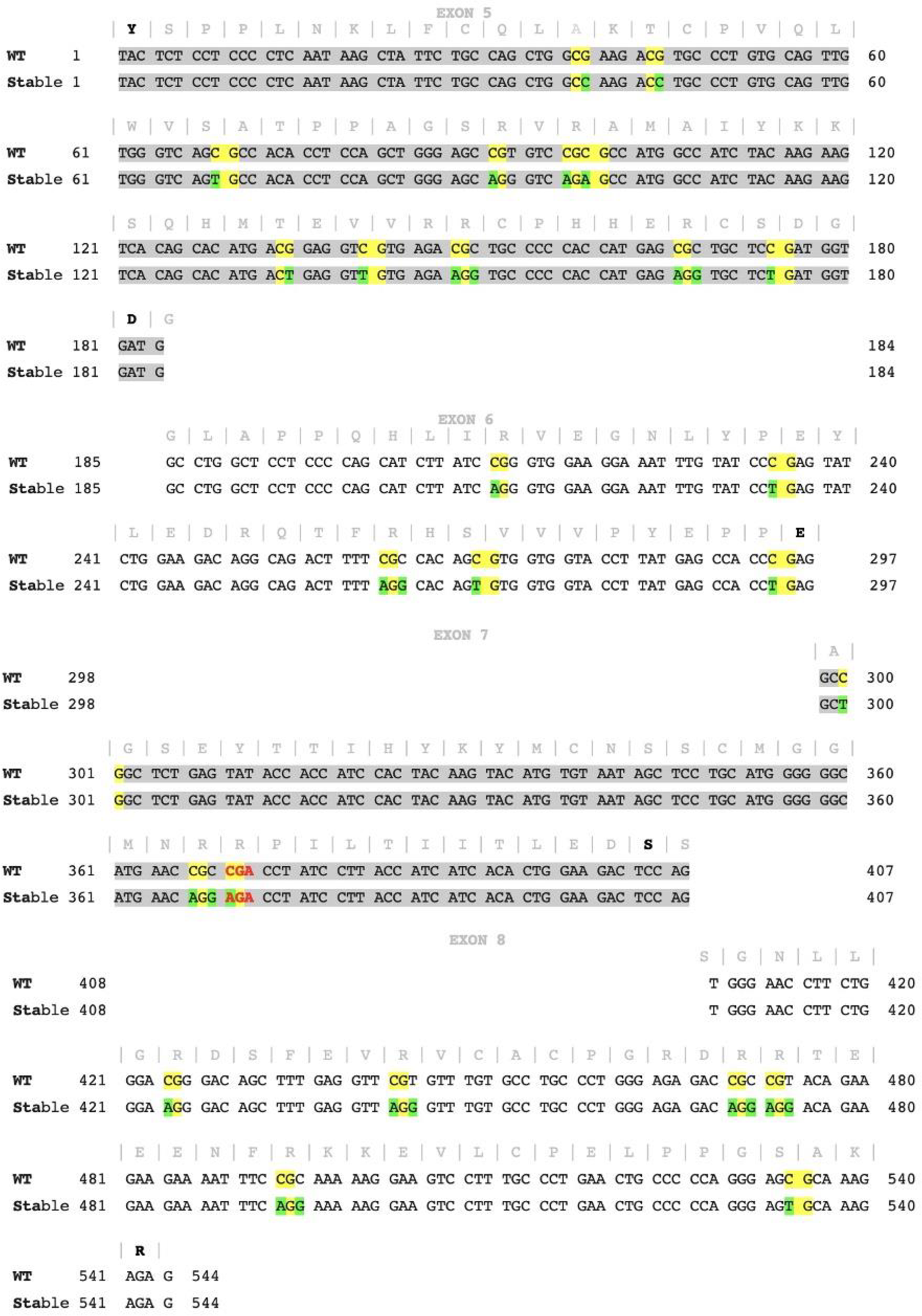

Supplement: S3 Fig — 22 CpG sites highlighted in yellow are replaced as shown by synonymous green-highlighted bases. (TIF) [file pone.0284327.s003.tif]

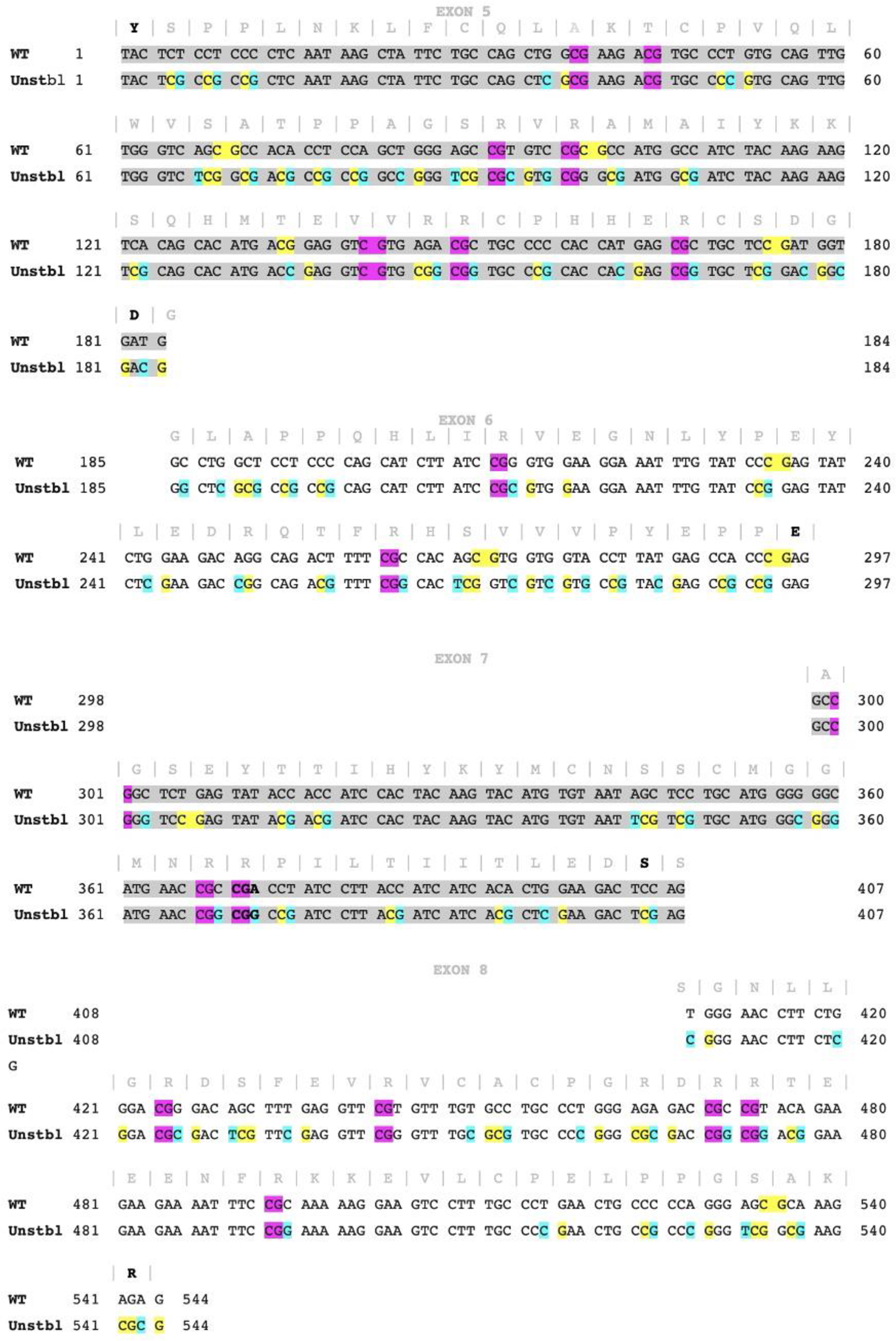

Supplement: S4 Fig — In addition to the retained wild-type CpG sites (highlighted in purple), 72 new CpG sites are created by synonymous replacement by the bases highlighted in either blue or yellow. (TIF) [file pone.0284327.s004.tif]

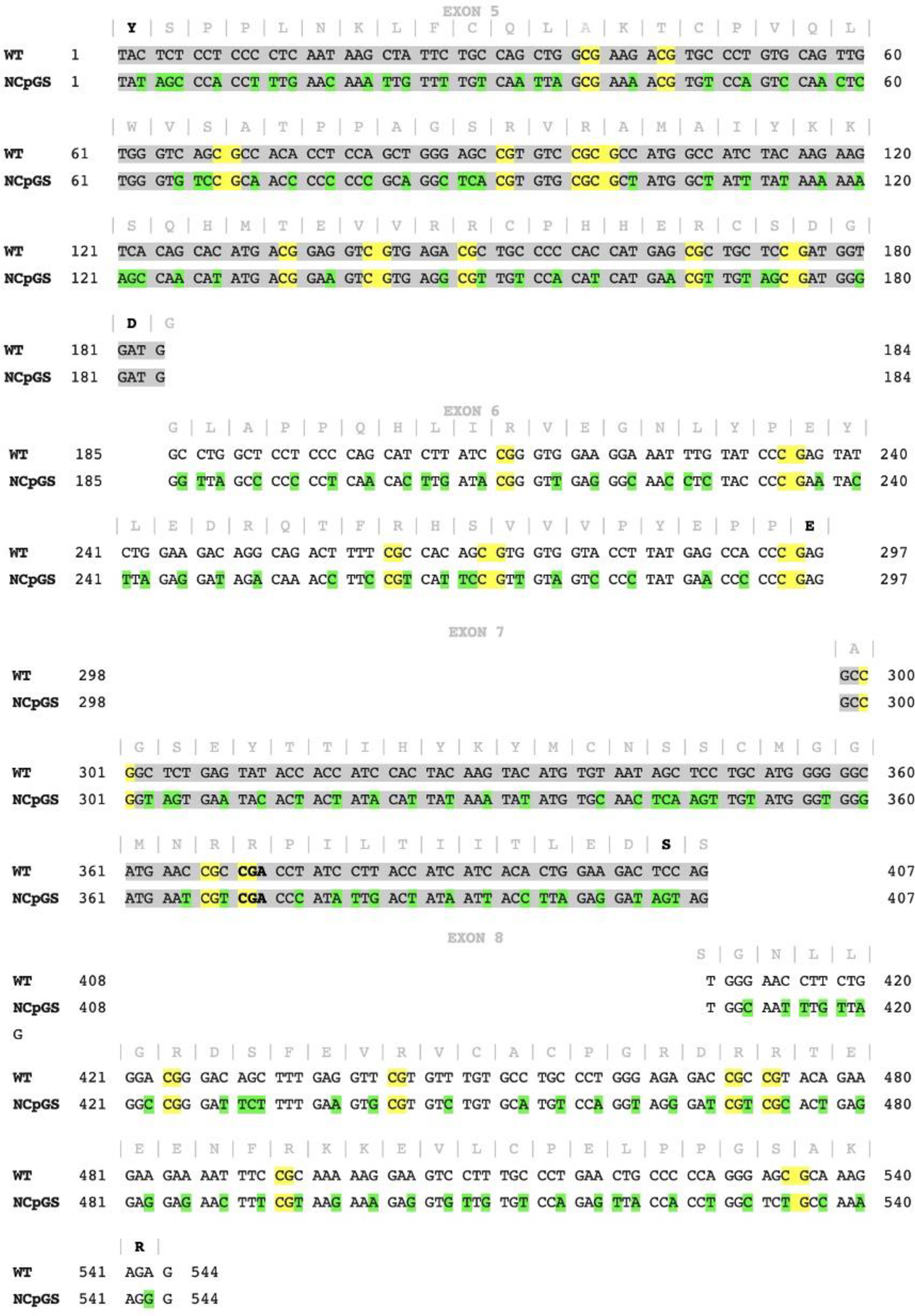

Supplement: S5 Fig — CpG sites highlighted in yellow remain conserved in both sequences. Green-highlighted bases in the NCpGS sequence represent all other (181) possible synonymous nucleotide changes. (TIF) [file pone.0284327.s005.tif]

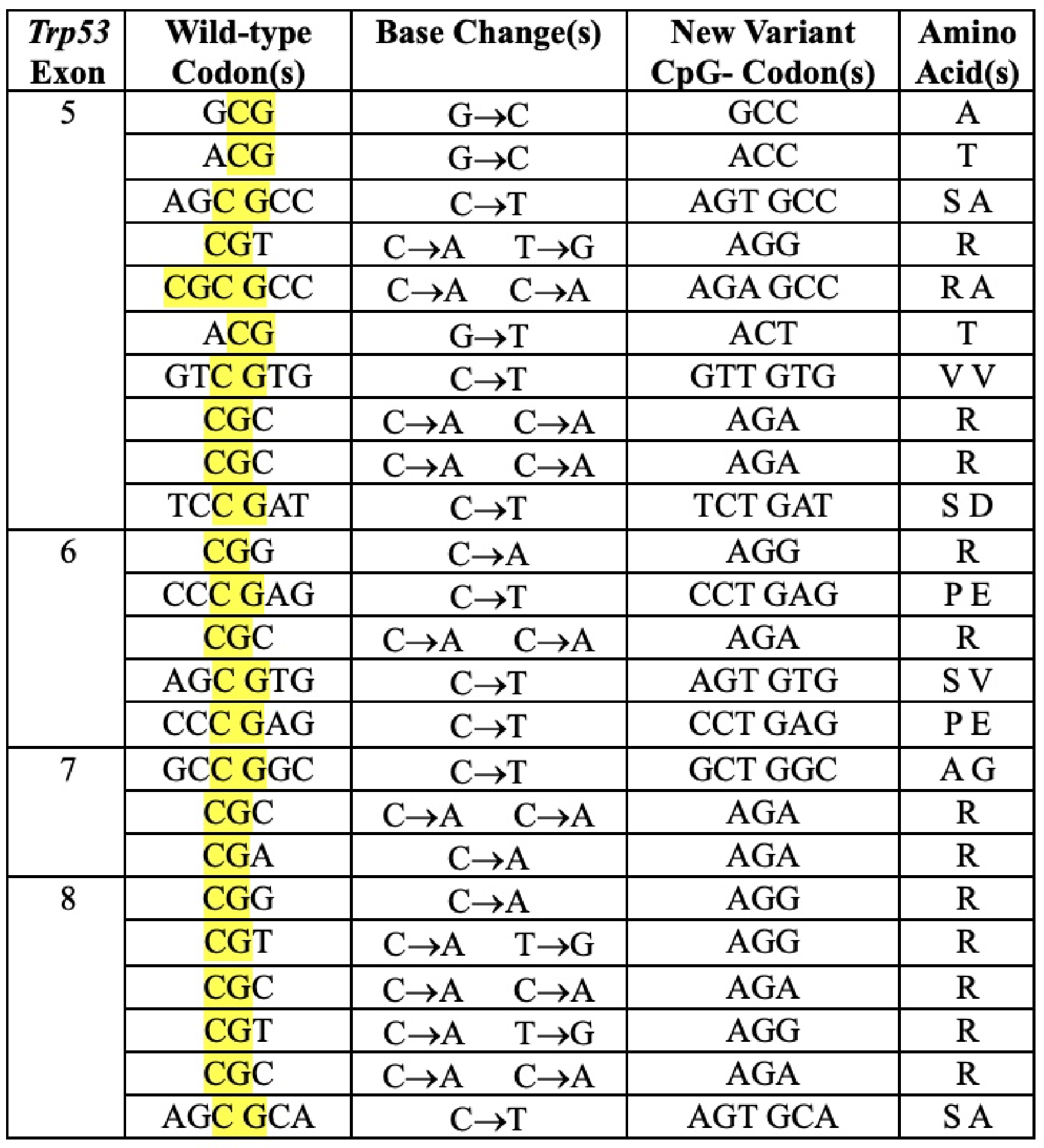

Supplement: S1 Table — CpG sites are highlighted. (TIF) [file pone.0284327.s006.tif]

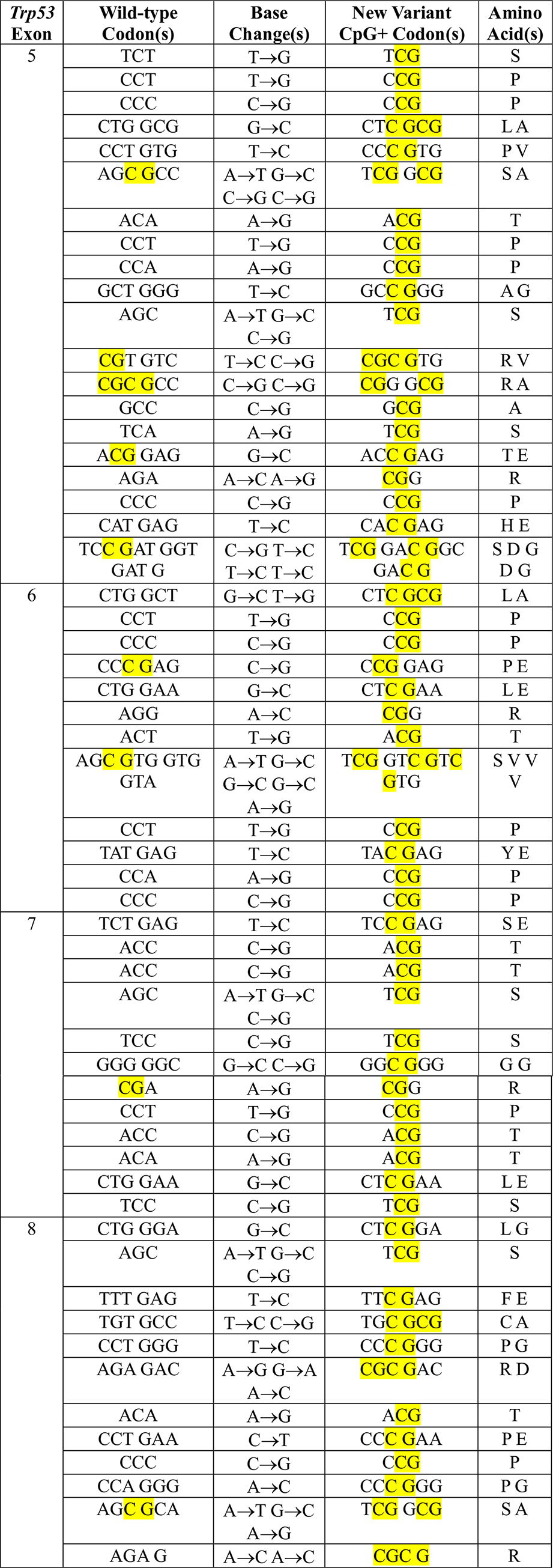

Supplement: S2 Table — CpG sites are highlighted. (TIF) [file pone.0284327.s007.tif]
