# Supplementary material for: Synonymous alterations of cancer-associated Trp53 CpG mutational hotspots cause fatal developmental jaw malocclusions but no tumors in knock-in mice
Source: PLoS One. 2023 Apr 13;18(4):e0284327. doi: 10.1371/journal.pone.0284327 (PMC10101519; doi:10.1371/journal.pone.0284327)
Supplement: Attachment — Submitted filename: Response to Reviewers.docx [file pone.0284327.s008.docx]

Response to Reviewers

1. Please ensure that your manuscript meets PLOS ONE's style requirements, including those for file naming. The PLOS ONE style templates can be found at 
<https://journals.plos.org/plosone/s/file?id=wjVg/PLOSOne_formatting_sample_main_body.pdf> and 
<https://journals.plos.org/plosone/s/file?id=ba62/PLOSOne_formatting_sample_title_authors_affiliations.pdf>

Au: This has been done (please note that EndNote PLOS ONE style automatically includes the PMID and PMCID codes in the citation – please delete if unnecessary).

2. In your Methods section, please provide additional information on the animal research and ensure you have included details on : (1) methods of sacrifice (2) methods of anesthesia and/or analgesia, and (3) the cause of death for animals before they meet the criteria for euthanasia.

Au: Thank you, this has been done, and now includes the following wording:

*Mice in this study were monitored weekly for weight changes, signs of discomfort, or poor health; where humanely indicated by such endpoints (e.g., signs of pain, inability to reach food, or ≥ 20% weight loss), mice were sacrificed by carbon dioxide asphyxiation. No anaesthesia or analgesia was carried out. Pre-euthanasia causes of death are listed in Table 1.*

3. Thank you for stating the following in the Acknowledgments/ Funding Section of your manuscript:

This work was supported by a donation from the family of Mr Frank Wolf to the St Vincent’s Curran Foundation. The funders had no role in study design, or in manuscript preparation.

We note that you have provided funding information that is not currently declared in your Funding Statement. However, funding information should not appear in the Acknowledgments section or other areas of your manuscript. We will only publish funding information present in the Funding Statement section of the online submission form. 
Please remove any funding-related text from the manuscript and let us know how you would like to update your Funding Statement. Currently, your Funding Statement reads as follows:

The funders had no role in study design, data collection and analysis, decision to publish, or preparation of the manuscript.

Please include your amended statements within your cover letter; we will change the online submission form on your behalf.

Au: Thank you for clarifying this. As instructed, the Funding Statement within the manuscript has now been removed. The Cover Letter now requests inserting the following revised wording in the online Funding Statement:

*This work was supported by a donation from the family of Mr Frank Wolf to the St Vincent’s Curran Foundation. The funders had no role in study design, data collection and analysis, decision to publish, or preparation of the manuscript*.

4. Your ethics statement should only appear in the Methods section of your manuscript. If your ethics statement is written in any section besides the Methods, please delete it from any other section.

Au: Thank you, again, for clarifying this. The additional Ethics Statement has been deleted.

5. Please include captions for your Supporting Information files at the end of your manuscript, and update any in-text citations to match accordingly. Please see our Supporting Information guidelines for more information: <http://journals.plos.org/plosone/s/supporting-information>.

Au: Thank you. This has been done.

6. Please review your reference list to ensure that it is complete and correct. If you have cited papers that have been retracted, please include the rationale for doing so in the manuscript text, or remove these references and replace them with relevant current references. Any changes to the reference list should be mentioned in the rebuttal letter that accompanies your revised manuscript. If you need to cite a retracted article, indicate the article’s retracted status in the References list and also include a citation and full reference for the retraction notice.

Au: The reference list is complete. The additional references proposed by the Reviewer have been added, and other added references mentioned below in the rebuttal section.

5. Review Comments to the Author

Reviewer #1: The paper describes a interesting experiment: the authors substituted the DNA-binding exons 5-8 of Trp53, the mouse ortholog of human TP53, with variant-CpG (either CpG-depleted or -enriched) sequences predicted to encode the normal p53 amino acid sequence. It is important that a control construct was also created in which all non-CpG sites were synonymously substituted. I do not see any need in additional experiments (many of them can be suggested but I am sure that the paper represents a solid publishable unit).

Comments:
The authors suggested that " that conserved CpG sites exert non-coding phenotypic effects via pre-translational cis-interactions of 5-methylcytosine with methyl-binding proteins which regulate mRNA transcript initiation, expression or splicing". May be, may be not. I think that other possible explanations should be discussed (the discussion is too short anyway). For example, a change of two tandem CpG containing codons of SARS-CoV-2 (that employs the host translation system substantially change properties of the spike protein most likely due to changes in translation :

The Functional Consequences of the Novel Ribosomal Pausing Site in SARS-CoV-2 Spike Glycoprotein RNA.
Postnikova OA, Uppal S, Huang W, Kane MA, Villasmil R, Rogozin IB, Poliakov E, Redmond TM. Int J Mol Sci. 2021 Jun 17;22(12):6490.

or secondary structure effects:

Role of mRNA structure in the control of protein folding. Faure G, Ogurtsov AY, Shabalina SA, Koonin EV. Nucleic Acids Res. 2016 Dec 15;44(22):10898-10911.

Something brief, the paper will benefit, I am sure.

Au: Thank you for this constructive criticism. My colleagues and I fully agree with the above points, and with the observation that the Discussion as it stands is not optimally detailed. As a result of these comments, the following changes have been made:

- We have included the following new paragraph in the Discussion:

*It should be noted that there are additional explanations for these results which are independent of MBPs. For example, one possibility is that alterations of intragenic CpG sites could create or remove as-yet-unrecognised alternative transcription initiation sites that in turn alter function of the encoded protein [89, 90]. A second possibility is that gain or loss of CpG sites alters mRNA secondary structure and (hence) protein folding [91], perhaps via changes in base stacking and Z-RNA formation [92, 93] due to modification of CpG step number or position [94, 95]. Yet another possibility is that altered intragenic CpG content could affect ribosomal translational pausing with consequent downstream effects on protein translation efficiency or subdomain folding [96].*

- These points are supported by the studies referenced by the Reviewer, as well as a further six supportive citations, which raise the same or similar interpretations.
- Given the improvement made by this new section, we have also improved the final sentence of the original Discussion by adding three references which note that only a single gene or protein need be affected to create the observed dental phenotype.
